# Comparison of flexible, open with semi-rigid, closed annuloplasty-rings for mitral valve repair

**DOI:** 10.1186/s13019-021-01405-1

**Published:** 2021-03-20

**Authors:** Ayse Cetinkaya, Maryam Waheed, Karin Bramlage, Oliver Johannes Liakopoulos, Mohamed Zeriouh, Stefan Hein, Peter Bramlage, Markus Schönburg, Yeong-Hoon Choi, Manfred Richter

**Affiliations:** 1 Department of Cardiac Surgery, Kerckhoff-Heart Center Bad Nauheim, Campus of the University Hospital Giessen, Justus-Liebig Universiy Giessen, Benekestraße 2-8, 61231 Bad Nauheim, Germany; 2 Institute for Pharmacology and Preventive Medicine, Cloppenburg, Germany

**Keywords:** Mitral valve repair, Mitral regurgitation, Annuloplasty, Open ring, Closed ring

## Abstract

**Background:**

Mitral regurgitation is a frequent valvular disease, with an increasing prevalence. We analysed the long-term outcomes of mitral valve repair procedures conducted over the last 10 years in our clinic using almost exclusively two different annuloplasty ring types.

**Methods:**

A single-centre, retrospective analysis of mitral valve surgeries conducted between January 2005 and December 2015 for patients undergoing first-line mitral valve repair with either open (Cosgrove) or closed (CE Physio / Physio II) annuloplasty (OA or CA, respectively) rings.

**Results:**

In total, 1120 patient documentations were available of which 528 underwent OA and 592 patients CA. The median age of patients was 64.0 years and 41.1% were female. The majority of these patients underwent the procedure because of degenerative valve disease. Rates of successful repair were about 90%, 72 h procedural mortality was 0.6% and the rate of re-intervention was 0.6% within the first 30 days. Functional (mitral regurgitation, left ventricular ejection fraction, left ventricular end-diastolic and systolic diameter and New York Heart Association class) as well as hard outcomes were comparable. 77.7 and 74.4% of patients were alive at the 10-year follow-up in the OA and CA groups, respectively. Upon multivariable adjustment, the hazard ratio was 0.926 (95% CI: 0.642–1.3135; *p* = 0.681).

**Conclusions:**

The functional outcome and survival rates up to 10 years after mitral valve repair were comparable using open and closed annuloplasty rings. Whether this means these rings are interchangeable or a carefully selection of the best-for-the-patient devices will be subject of future investigations.

## Background

Primary mitral regurgitation (MR) is the result of pathology affecting at least one component of the mitral valve apparatus and is usually the consequence of degenerative disease. Surgical intervention is associated with high repair rates and low operative morbidity and mortality [1]. Current international guidelines all advise mitral valve repair (MVR) for symptomatic patients with severe MR. [2, 3]

MR occurs because the two leaflets normally involved in sealing the mitral valve to retrograde flow may not coapt properly. MVR, therefore, comprises restoration of leaflet coaptation and the implantation of standardised annuloplasty rings [4, 5]. Annuloplasty has been shown to improve valve repair durability and stabilise the entire posterior mitral annulus [6, 7]. Although various annuloplasty devices are available including flexible vs. semi-rigid vs. rigid, incomplete vs. complete, planar vs. saddle-shaped and adjustable vs. non-adjustable, there is a lack of sufficiently powered data on the relative merits of each ring design [4, 5, 8, 9].

It was for this reason that we retrospectively gathered the patient and procedural characteristics of patients undergoing mitral valve repair in our clinic. As we almost exclusively use only two different ring types, we focused our analysis on the flexible, C-shaped open *Cosgrove Edwards* and the semi-rigid closed *Carpentier-Edwards Physio / Physio II* annuloplasty rings. The aim of this analysis was to compare the long-term outcomes.

## Patients and methods

This study is a single-centre, retrospective analysis of mitral valve surgeries performed between January 2005 and December 2015. The study was approved by the site’s ethical committee and complied with the Declaration of Helsinki. Written, informed consent was not required due to the use of anonymised data already collected as part of routine practice.

### Patient population

Patients undergoing mitral valve repair were included. Patients had to undergo annuloplasty ring implantation using either the flexible, C-shaped open Cosgrove Edwards flexible band (termed open annuloplasty or OA group) or the semi-rigid closed Carpentier-Edwards Physio / Physio II device (termed closed annuloplasty or CA group). Both devices (OA vs. CA) were used by all surgeons involved depending on the clinical situation. While there is a potential preference of either device type by surgeon, the degree of which was not documented. Potential concomitant procedures were tricuspid valve reconstruction, ablation of AF, left atrial appendage closure and ASD or PFO closure. Patients undergoing first-line mitral valve replacement, patients receiving other ring types and those undergoing concomitant coronary artery bypass graft or aortic valve surgery were excluded. Follow-up data concerning complications and echocardiographic parameters were collected at the patient’s last FU visit.

### Endpoints of interest

We defined the following endpoints of interest for our analysis: 1) Survival, 2) freedom from reoperation, 3) the degree of postoperative MR, 4) the postoperative functional status based on the NYHA class, 5) the postoperative left ventricular ejection fraction (LVEF), and 6) the postoperative left ventricular enddiastolic diameter (LVEDD) / left ventricular endsystolic diameter (LVESD).

### Statistics

Data were analysed using descriptive statistics, with categorical variables presented as absolute values and frequencies (%) and the continuous variables presented as the median and interquartile range (IQR). Comparisons between the OA and CA groups were carried out using Mann-Whitney U-test for continuous variables and a Fisher’s exact or Chi-square test for categorical variables. Odds ratios (OR) were calculated by logistic regression and adjusted for the key baseline variables age, pulmonary hypertension, prior aortic valve replacement (AVR), LVEDD, LVESD, left/right atrium (mm), chordae elongation and logistic EuroSCORE. Survival analysis data was presented as Kaplan-Meier curves. In addition, hazard ratios (HR) were calculated by COX-regression and again adjusted for differences in the key baseline variables named above. In all multivariable analyses, only data of patients with available values for all variables taken into account for adjustment were included.

A two-tailed *p*-value of < 0.05 was considered statistically significant. All statistical tests were performed using IBM SPSS Statistics software version 24.0 (IBM Corporation, Armonk, NY, USA).

## Results

**Fig. 1:**
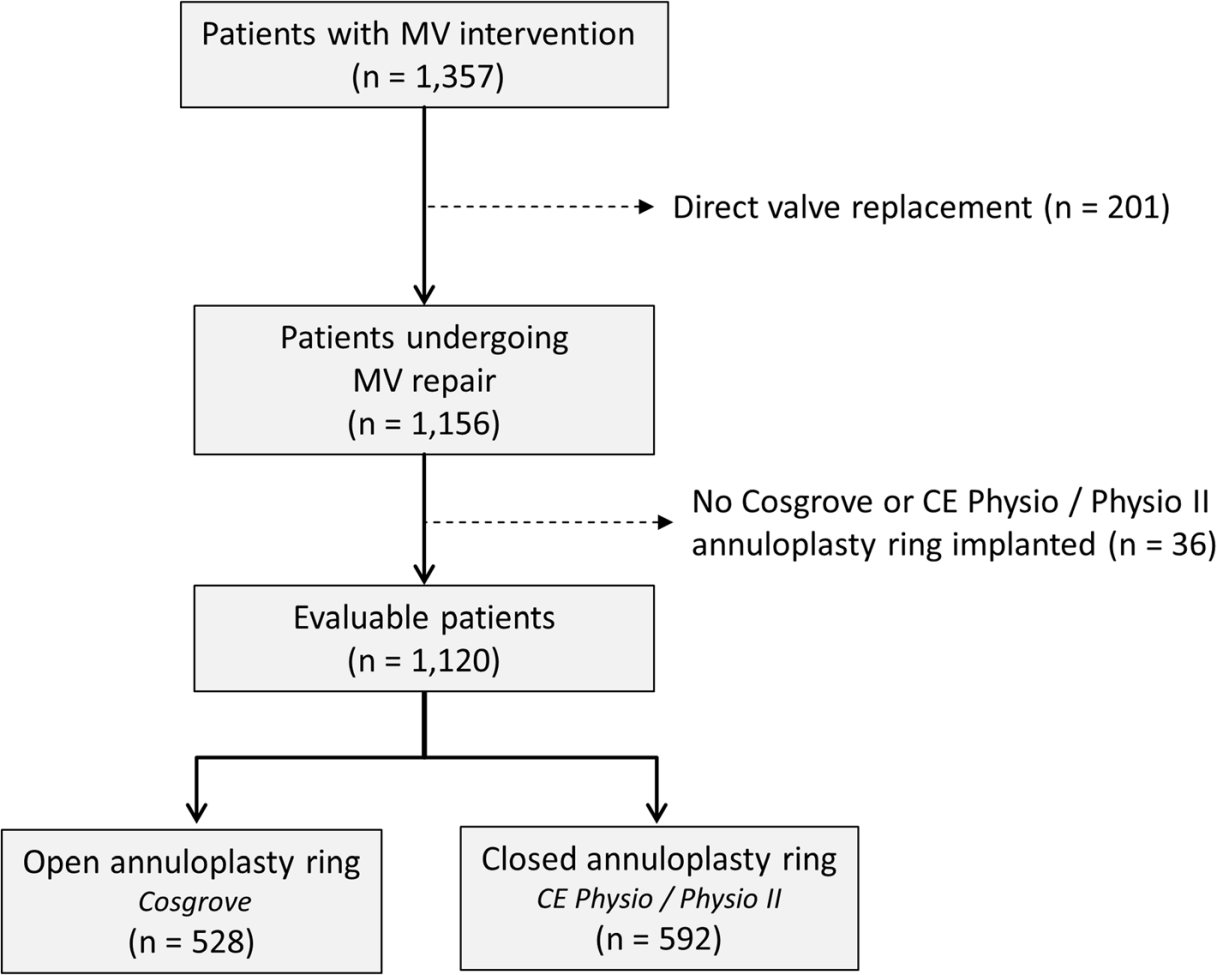
Flow chart outlining study patient cohorts. *Legend:* none

A total of 1357 patients were documented. Patients undergoing direct MVR (*n* = 201) and those receiving other annuloplasty rings than the Cosgrove-Edwards or the Carpentier-Edwards Physio/Physio II (*n* = 36) as part of their MVR were excluded. Of the remaining 1120 patients 528 were assigned to the OA group and 592 patients to the CA group (Fig. 1).

### Patient characteristics

Patients had a median age of 64.0 years and 41.1% were female (Table 1). Hypertension was a frequent risk factor (53.1%); diabetes (7.1%) and COPD (11.1%) were frequent comorbid conditions. Disease conditions associated with a causal relationship to mitral valve (MV) disease were pulmonary hypertension (10.0% of patients) and AF (29.7%). Overall, 73.6% of patient had NYHA III/IV defined symptoms. The median logistic EuroSCORE was 3.3%.

**Table 1:** Patient characteristics

	Total ***N*** = 1120 ^a^	OA ***N*** = 528 ^a^	CA ***N*** = 592 ^a^	p-value OA vs. CA
	% or Median [IQR]	% or Median [IQR]	% or Median [IQR]	
Age in years	64.0 [54.0–73.0]	66.0 [55.0–74.0]	63.0 [54.0–72.0]	0.060
Female gender, %	41.1	42.2	40.0	0.455
BMI (kg/m^2^)^b^	25.8 [23.1–28.7]	26.1 [23.5–29.0]	25.6 [22.9–28.4]	**0.035**
CV risk factors				
Hypertension, %	53.1	52.5	53.7	0.675
Dyslipidemia, %	16.0	15.0	16.9	0.386
Comorbidities general				
Diabetes mellitus, %	7.7	8.3	7.1	0.437
Creatinine (mg/dL)	0.9 [0.7–1.0]	0.9 [0.7–1.0]	0.9 [0.7–1.1]	0.773
Kidney failure (Crea. > 2.26 mg/dL), %	1.6	2.3	1.0	0.094
Stroke, %	4.2	4.9	3.5	0.251
COPD, %	11.1	11.0	11.1	0.931
PAD, %	2.4	3.0	1.9	0.202
Comorbidity cardiac				
AF, %	29.7	28.1	31.2	0.257
CAD, %	8.7	8.5	8.8	0.877
Prior MI (≤ 90 days), %	0.4	0.4	0.5	1.000
Prior CABG, %	2.9	2.8	2.9	0.975
Prior aortic valve replacement, %	1.6	2.5	0.8	**0.032**
Prior pacemaker, %	1.6	1.5	1.7	0.815
NYHA class III/IV, %	73.6	75.1	72.3	0.281
CCS class III/IV, %	3.8	4.7	3.0	0.141
Pulmonary hypertension, %	10.0	11.6	8.6	0.104
Emergency indication for surgery, %	1.8	2.1	1.5	0.482
Log EuroSCORE I, %	3.3 [1.6–7.1]	3.5 [1.7–8.0]	3.0 [1.5–6.6]	**0.032**

^a^As data are largely complete, e.g., maximum of 3 out of 1120 variables missing for single variables we omitted to name the number of patients available for each variable

^b^ For BMI there is only data for 896 patients (418 with OA/ 478 with CA)

*AF* atrial fibrillation, *BMI* body mass index, *CABG* coronary artery bypass graft, *CAD* coronary artery disease, *CCS* Canadian Cardiovascular Society, *COPD* chronic obstructive pulmonary disease, *Crea.* Creatinine, *CV* cardiovascular, *MI* myocardial infarction, *NYHA* New York Heart Association

More patients in the OA group had prior aortic valve replacement (2.5 vs. 0.8%; *p* = 0.032) and a higher logistic EuroSCORE I (3.5 vs. 3.0%; p = 0.032) (Table 1).

### Mitral valve surgery

The majority of patients in both groups underwent mitral valve repair because of a degenerated valve (92.6 in the OA vs. 93.5% in the CA groups; *p* = 0.523) (Table 2). There were echocardiographic differences between groups including a lower left (median 52.0 vs. 55.0 mm; *p* = 0.002), right atrial diameter (45.0 vs. 47.0 mm; *p* = 0.016) and a reduced mitral opening (median 3.7 vs. 4.1 mm; *p* = 0.021) in the OA group.

**Table 2:** MV pathology and echocardiographic parameters and procedural details

	OA (n = 528)	CA (n = 592)	
	%	%	p-value
**MV pathologies**			
Functional	7.4	6.4	0.523
Degenerative	92.6	93.5	
Acute endocarditis	2.5	1.9	0.486
Annulus dilatation	94.9	96.6	0.148
Annulus calcification	3.6	4.1	0.692
AML prolapse	19.1	23.6	0.066
AML flail	5.1	6.8	0.247
PML prolapse	71.4	68.9	0.365
PML flail	51.7	46.6	0.089
Chordae elongation	22.5	28.4	**0.025**
Restrictive leaflet	3.0	3.4	0.742
MV stenosis	0.6	0.3	0.671
MV insufficiency ≥ grade II	99.6	99.3	0.690
**Echocardiographic parameters**	**Median [IQR]**	**Median [IQR]**	
LVEF, %	60.0 [55.0–63.0]	60.0 [55.0–64.0]	0.660
LVEDD (mm)	55.0 [51.0–59.0]	56.0 [52.0–60.0]	**0.003**
LVESD (mm)	35.0 [31.0–39.0]	36.0 [32.0–41.0]	**0.001**
Left atrial diameter (mm)	52.0 [46.0–59.0]	55.0 [47.0–63.0]	**0.002**
Right atrial diameter (mm)	45.0 [38.0–52.0]	47.0 [39.0–53.0]	**0.016**
Mitral opening (mm)	3.7 [3.1–4.6]	4.1 [3.4–4.8]	**0.021**
PISA radius (mm)	1.0 [1.0–1.3]	1.2 [1.0–1.4]	0.128
Vena contracta (mm)	7.0 [5.0–8.0]	7.0 [5.0–7.0]	0.397
**Procedural details**	**%**	**%**	
Operative approach			**< 0.001**
MIC	59.1	71.3	
CS	40.9	28.7	
Mitral valve repair			
Triangular resection PML	16.7	15.7	**0.664**
Quadr. resection PML	23.3	12.3	**< 0.001**
AML reconstruction	12.3	22.6	**< 0.001**
PML reconstruction	75.4	72.1	0.218
Neo chordae AML	8.9	15.2	**0.001**
Neo chordae PML	29.9	35.3	0.056
Cleft plicatur	14.2	28.2	**< 0.001**
Annuloplasty ring size			**< 0.001**
26–28	36.4	15.4	
30–32	40.4	38.9	
34–36	20.9	39.1	
38–40	2.3	6.4	
Concomitant procedures			
Cryoablation	25.8	28.7	0.276
LAA closure	26.7	24.0	0.296
Concomitant TVR	14.8	12.0	0.168
PFO closure	12.5	6.4	**< 0.001**
ASD closure	0.9	1.0	0.910
Myxom	0.6	0.3	0.671
Times	**Median [IQR]**	**Median [IQR]**	
Procedure time (min)	185.0 [164.0–215.0]	195.0 [171.0–230.8]	**< 0.001**
CPB time (min)	116.0 [97.0–140.0]	128.0 [106.3–156.0]	**< 0.001**
x-clamp time (min)	75.0 [63.0–94.0]	79.0 [64.0–100.0]	0.074
Length of intubation (h)	10.0 [8.0–14.0]	10.0 [8.0–13.0]	0.905
Length of ICU (h)	24.0 [22.0–47.0]	24.0 [21.0–48.0]	0.686
Length of hospital stay (d)	10.0 [8.0–13.0]	10.0 [8.0–13.0]	0.903
	**%**	**%**	
Rate of successful repair ^a^	88.4	90.5	0.253
MV replacement after repair failure	11.0	9.1	0.299
Conversion to sternotomy	2.6	1.9	0.537

*AML* anterior mitral valve leaflet, *ASD* atrial septal defect, *CPB* cardiopulmonary bypass, *CS* conventional sternotomy, *ICU* intensive care unit, *IQR* interquartile range, *LAA* left atrial appendage closure, *LVEDD* left ventricular enddiastolic diameter, *LVEF* left ventricular ejection fraction, *LVESD* left ventricular endsystolic diameter, *MIC* minimally invasive mitral valve surgery, *MV* mitral valve, *PFO* patent foramen ovale, *PISA* proximal isovelocity surface area, *PML* posterior mitral valve leaflet, *TVR* tricuspidal valve repair

^a^5 patients were removed as they died within 72 h after the intervention (electromechanical decoupling n = 1, right ventricular failure n = 1, low cardiac output n = 2, cardiogenic shock and kidney failure *n* = 1)

Fewer patients receiving OA had minimal invasive valve surgery (*p* < 0.001) and AML reconstruction (p < 0.001) and the inserted annuloplasty rings generally had a smaller size (Table 2). PML quadrangular resection was more common with OA (23.3% vs. 12.3%; p < 0.001), while neo-chordae AML were more common with CA (15.2% vs. 8.9%; *p* = 0.001) as was cleft plicature (28.2% vs. 14.2%; p < 0.001). Concomitant procedures (including atrial fibrillation (AF) cryoablation, left atrial appendage (LAA) closure and tricuspid valve repair) were comparable in both groups. The number of patients requiring patent foramen ovale (PFO) closures, however, was higher in the OA group. The cardio-pulmonary bypass time as well as the overall procedure time was shorter in the OA group.

There was a high rate of successful repair reaching 88.4% in the OA and 90.5% in the CA group (*p* = 0.253). Only 8 patients undergoing minimal invasive surgery in either group were converted to median sternotomy (2.2%). Pleural effusion was 1.7% in the OA group and 2.7% in the CA group multivariable adjusted OR 0.34 (95%CI: 0.12–0.99).

#### Functional outcomes

**Fig. 2:**
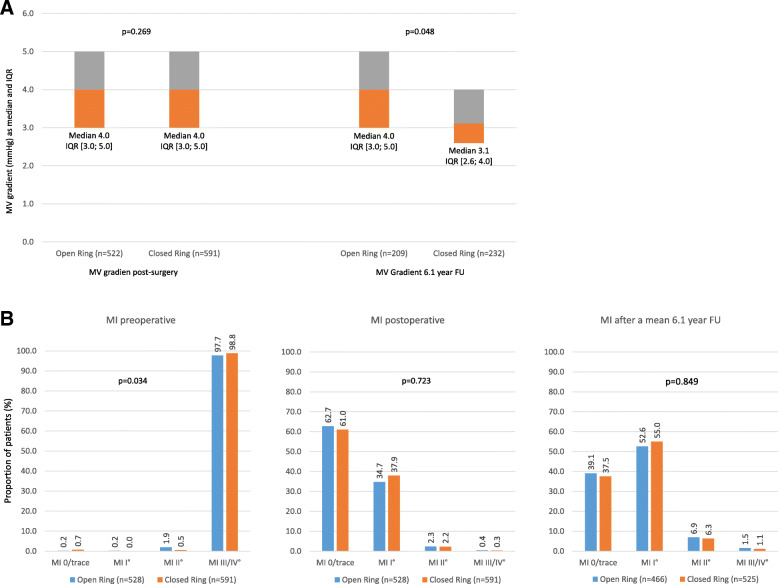
Mitral valve gradient and competency. *Legend:* none

The median MV gradient in patients receiving an OA was 4.0 at both the baseline and follow-up (FU) visit, whereas it was reduced from 4.0 to 3.1 at FU in patients with CA. Thus, the median gradient was significantly different between both groups at the mean 6.1 year FU (*p* = 0.048) (Fig. 2a) which was a potential result of greater ring sizes in the CA group (*p* < 0.001). The proportion of patients with II° or III°/IV° grade mitral insufficiency was strongly reduced after the intervention and at 6.1 years with no difference between the OA and CA patient groups (Fig. 2b). Postoperative Systolic Anterior Motion was observed in 52 out of 1120 patients with no statistically significant difference between groups (OA 4.4% vs. CA 4.9%; *p* = 0.667). There was, however, a noteworthy trend with higher rates of I° degree mitral insufficiency at 6.1 years FU compared with the immediate postoperative period.

**Fig. 3:**
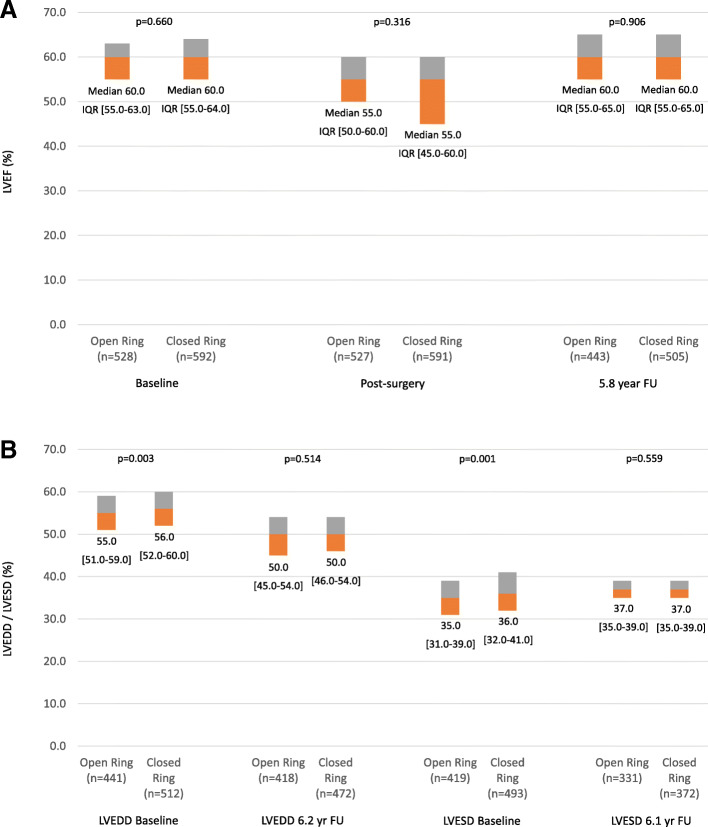
Left Ventricular Dimension and Function. *Legend:* none

The LVEF dropped from a median baseline value of 60% (e.g. pre-intervention) to a median LVEF of 55% post-procedurally. There were no differences in the LVEF at baseline (*p* = 0.660) or after the intervention (*p* = 0.316) between groups. LVEF recovered later on (5.8 years) back to the baseline value of 60% which was identical in both groups (*p* = 0.906) (Fig. 3a). LVEDD was 55 mm in the OA group and 56 mm in the CA group at baseline and 50 mm in both groups at the follow-up. While their difference at baseline was statistically significant (*p* = 0.003), it was not at the follow-up (Fig. 3b). LVESD was 35 mm and 36 mm, respectively at baseline (*p* = 0.001) and 37 mm at the follow-up in either group (*p* = 0.559).

**Fig. 4:**
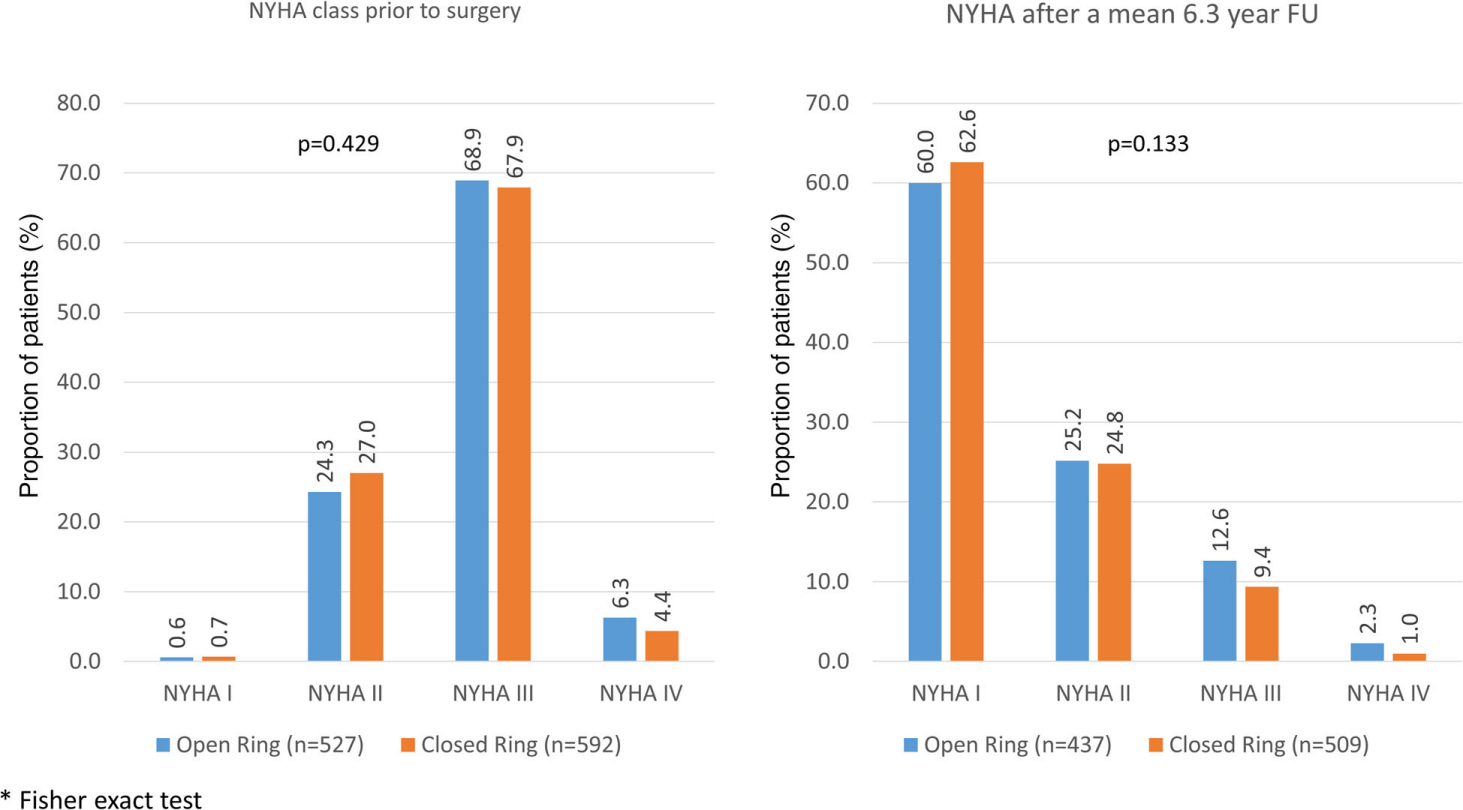
NYHA class. *Legend:* none

In agreement with the decrease in mitral valve insufficiency at a preserved LVEF, there was a considerable improvement of symptoms observed with the majority of patients being NYHA I or II at 6.3 years (Fig. 4) with no difference between groups (*p* = 0.133).

#### Hard outcomes

The rates of re-intervention following the immediate post-procedural phase and up to 30 days was 0.6% in the OA and 0.5% in the CA group (adjusted OR 0.97; 95%CI 0.17 to 5.47).

**Fig. 5:**
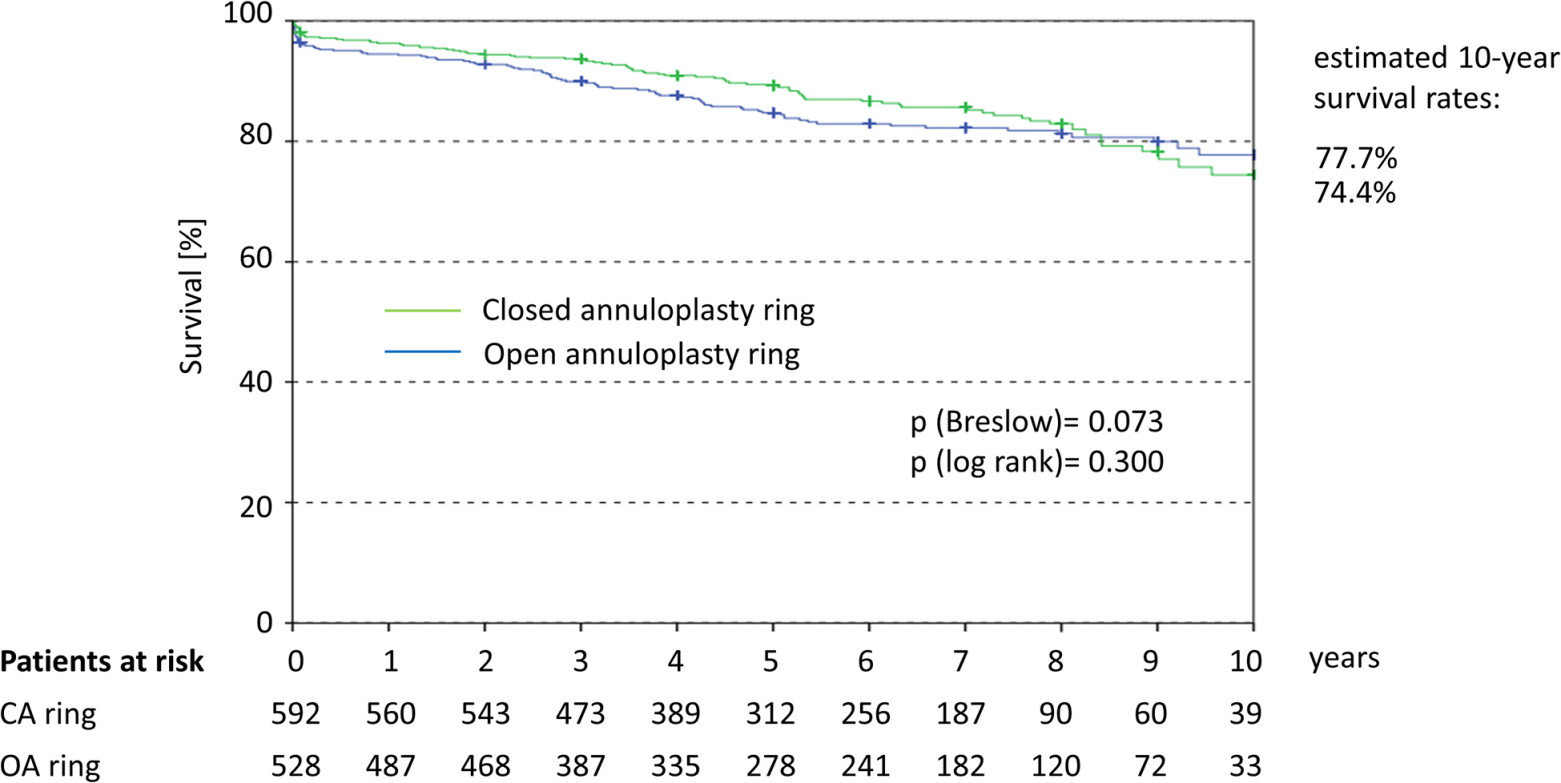
Kaplan-Meier curve for the long-term survival after MVR. *Legend:* HR calculated by COX regression was 1.171 (95%CI 0.868–1.580; *p* = 0.300). After adjustment for age, pulmonary hypertension, prior AVR, LVEDD, LVESD, left/right atrium (mm), chordae elongation and log EuroSCORE the HR was 0.926 (95%CI 0.642–1.3135 *p* = 0.681) in favour of closed annuloplasty ring. In the multivariate Cox analysis only data of patients with available values for all variables taken into account for adjustment were included (*n* = 820)

Immediate within 72 h procedural mortality affected 7 out of 1120 patients (0.6%). Outcomes at 30 days showed comparable rates for all-cause death, stroke, acute renal failure, MI and pacemaker between groups (Table 3). An initial significant difference in the rate of non-CV death disappeared upon adjustment for differences in baseline characteristics (adjusted OR 5.00; 95%CI 0.52–48.28). Long-term mortality was comparable between patients in the OA and CA groups, with an estimated 10-year survival of 77.7 and 74.4% for patients, respectively (log rank *p* = 0.300; adj. HR 0.926 (95%CI: 0.642–1.3135; *p* = 0.681) (Fig. 5).

**Table 3:** Procedure-related complications and 30-day outcomes

	OA (%) N = 528	CA (%) N = 592	Unadjusted OR 95% CI	Adjusted OR ^a^ 95% CI
**Procedure related complications**				
Immediate 72 h procedural mortality	0.9	0.3	2.82 (0.55 14.60)	1.42 (0.21–9.70)
MVI ≥ II post op	2.7	2.5	1.05 (0.50–2.19)	0.70 (0.28–1.73)
Wound infection	1.1	1.7	0.67 (0.24–1.85)	0.28 (0.06–1.38)
Pericardial tamponade	3.6	2.4	1.54 (0.77–3.11)	1.48 (0.58–3.77)
AV block grade III	6.3	5.4	1.17 (0.71–1.93)	0.98 (0.54–1.81)
Pneumonia	5.1	3.5	1.47 (0.82–2.62)	1.22 (0.59–2.54)
Pneumothorax	0.6	1.4	0.42 (0.11–1.58)	0.60 (0.15–2.48)
Pleural effusion	1.7	2.7	0.63 (0.27–1.43)	**0.34 (0.12–0.99)**
AF	16.9	15.7	1.01 (0.79–1.50)	1.05 (0.71–1.55)
**30-day outcomes**				
Death	3.6	2.0	1.80 (0.87–3.75)	1.16 (0.45–2.96)
Cardiovascular death	1.9	1.7	1.12 (0.46–2.72)	0.73 (0.24–2.24)
Non-CV death	1.7	0.3	**5.12 (1.10–23.78)**	5.00 (0.52–48.28)
Stroke	3.6	3.4	1.07 (0.56–2.02)	0.91 (0.43–1.89)
Acute renal failure	6.3	5.1	1.30 (0.78–2.17)	0.97 (0.49–1.91)
Myocardial infarction	0.4	0.7	0.56 (0.10–3.06)	0.66 (0.11–4.15)
Pacemaker implantation	6.8	6.3	1.10 (0.68–1.77)	0.91 (0.51–1.60)
Re- MV surgery	0.6	0.5	1.12 (0.22–5.58)	0.97 (0.17–5.47)

*AF* atrial fibrillation, *AV* atrioventricular, *CV* cardiovascular, *MV* mitral valve, *MVI* mitral valve insufficiency

^a^ORs were calculated by logistic regression and adjusted for age, pulmonary hypertension, prior AVR, LVEDD, LVESD, left/right atrium (mm), chordae elongation and log EuroScore. In the multivariate analysis only data of patients with available values for all variables taken into account for adjustment were included (*n* = 897). No BMI or mitral opening data were considered as there was a considerable degree of missing values

## Discussion

In our dataset of 1156 MVR procedures, we almost exclusively used the open *Cosgrove-Edwards* and the closed *Carpentier-Edwards Physio/Physio II* in patients with degenerative valve disease. Rates of successful repair were about 90%, 72 h procedural mortality was 0.6% and the rate of re-intervention was 0.6% within the first 30 days. Functional as well as hard outcomes with both ring types were largely comparable, with a slightly higher MV gradient seen with time in the CA group.

To the best of our knowledge, our dataset is the largest study with the longest follow-up so far comparing the use of open and closed annuloplasty rings for the treatment of MR. The majority of the patients experienced substantial improvement of their mitral insufficiency and symptom status. Interestingly, at 6.1 years, there was a slight worsening in the degree of mitral valve insufficiency with an increase in I° MR. It is reassuring though, that III° and IV° degree MR was not observed during our long-term follow-up giving confidence that the procedure achieved its clinical goals.

In an attempt to quantify potential differences between different annuloplasty rings, Khamooshian et al. performed a secondary data analysis [10]. They clustered the large number of more than 37 available rings into 3 groups of flexible, semi-rigid and rigid rings and focused on patients with either degenerative etiology or those patients with ischemic / dilated cardiomyopathy. They found that irrespective of the ring used, the LVEF remained unchanged after the intervention (just as in our analysis), the LVESD decreased with the use of all ring types, and LVEDD only decreased with the use of flexible and rigid rings, while there was no decrease associated with semi-rigid rings. We observed a decrease in both OA (with the flexible ring) and CA groups (with the semi-rigid ring), with no nominal difference between groups. The drop is unlike as in the Khamooshian analysis. LVESD was essentially unchanged at the follow-up.

A Pubmed search of the available literature over the last 10 years in June 2019, the keywords “annuloplasty, mitral, ring, comparison” resulted in a total of 41 references, of which seven actually presented a comparison of at least two different rings (1 animal model [11], 2 restricted to ischemic etiology [12–14] and 4 which did not specify the etiology [15, 16] as well as one computer-based modelling study [17]). The majority of these did not report on long-term functional and survival benefits, but focused on short-term hemodynamic results. Nishi [15] et al. found, that using the Cosgrove (*n* = 10), Sorin-Memo 3D (*n* = 17) and CE Physio II (*n* = 7) implantation rings, all controlled mitral regurgitation well, the Cosgrove more than the Sorin-Memo more than the CE Physio II had a dynamic diastolic to systolic change in mitral annular diameter during the cardiac cycle. Tsuneto [16] et al. reported from 31 patients, that the Cosgrove was found to be more flexible, while the Sorin-Memo ring maintained the elliptical shape more efficiently. Bouchez [18] et al. reported in a comparison of the CE Physio II (n = 17) with the Memo 3D (*n* = 16) that the mitral annulus dynamics after annuloplasty with the Physio II and Memo 3D rings demonstrated a better systolic 3D restoration of the saddle shape with the Physio II ring, whereas the saddle-shaped geometry improved significantly with the Memo 3D ring, as a dynamic phenomenon. The Memo 3D ring also showed increased anteroposterior annular mobility and folding dynamics throughout the cardiac cycle.

Silberman [13] et al. was the only to report on longer-term clinical outcomes in his report of 169 patients with ischemic MR. The symptom status (NYHA) was class III / IV in 33% with the flexible ring and 14% with the rigid ring (*p* = 0.03, MR grade was 1.15 and 0.7 (*p* = 0.006), respectively. There was no difference in LV function or dimensions. At follow-up, 29 patients (34%) in the flexible group had residual MR of moderate degree or greater compared with 6 (15%) in the rigid group (p = 0.03). Late mortality was observed in 32 out of 117 patients, which affected exclusively the flexible group (FU 58 months in the flexible and 14 months in the rigid group). Although these data on ischemic MR are reported on the same outcome, their quantity is hard to compare to our results as our database was exclusively built on degenerative mitral disease.

### Limitations

There were several limitations to this study. Firstly, due to its retrospective nature, data for certain fields may have been missing. Secondly, there were baseline differences between OA and CA groups, representing a possible source of bias. Thirdly, in the multivariate analyses, only data of patients with available values for all variables were taken into account for adjustment purposes (no imputation). Finally, major complications and echocardiography data were collected at the patient’s last FU visit – this data was not available for some patients as they only recently received their implant and it also excluded data for those patients lost to FU.

## Conclusions

In summary, our study showed that the use of OA and CA rings for the treatment of patients undergoing mitral valve repair were comparable, with both types of rings reducing mitral insufficiency and improving NYHA scores immediately after treatment, as well as at 30-days and ~ 6 years after treatment. Whether this means these rings are interchangeable or a carefully selection of the best-for-the-patient devices will be subject of future investigations.

## Data Availability

Data are available from the corresponding author upon reasonable request.

## Abbreviations

AF: Atrial fibrillation
AML: Anterior mitral valve leaflet
ASD: Atrial septal defect
AV: Atrioventricular
CA: Closed annulopasty
CABG: Coronary artery bypass graft
CAD: Coronary artery disease
CCS: Canadian Cardiovascular Society
COPD: Chronic obstructive pulmonary disease
CPB: Cardiopulmonary bypass
CS: Conventional sternotomy
CV: Cardiovascular
HR: Hazard Ratio
ICU: Intensive care unit
IQR: Interquartile range
LAA: Left atrial appendage
LVEDD: Left ventricular end-diastolic pressure
LVEF: Left ventricular ejection fraction
LVESD: Left ventricular end-systolic pressure
MIC: Minimally invasive mitral valve surgery
MV: Mitral valve
MVI: Mitral valve insufficiency
MVS: Mitral valve surgery
NYHA: New York Heart Association
OA: Open annulopasty
OR: Odds Ratio
PAD: Peripheral artery disease
PISA: Proximal isovelocity surface area
PFO: Patent foramen ovale
PML: Posterior mitral valve leaflet
TVR: Tricuspid valve repair
